# Raddeanin A inhibited epithelial-mesenchymal transition (EMT) and angiogenesis in glioblastoma by downregulating β-catenin expression

**DOI:** 10.7150/ijms.52206

**Published:** 2021-02-04

**Authors:** Bingshan Wu, Jianwei Zhu, Xingliang Dai, Lei Ye, Bin Wang, Hongwei Cheng, Weihong Wang

**Affiliations:** 1 Department of Neurosurgery, First Affiliated Hospital of Anhui Medical University, Hefei City, Anhui Province, China 230032.; 2 Department of Neurosurgery, Sichuan Provincial People's Hospital, University of Electronic Science and Technology of China, Chengdu City, Sichuan Province, 611731, China.

**Keywords:** Raddeanin A, EMT, angiogenesis, β-catenin, glioblastoma.

## Abstract

Raddeanin A (RA), an oleanane-type triterpenoid saponin derived from *Anemone raddeana* Regel, has been found to suppress the viability and metastasis of several cancers, including GBM, through various signaling pathways. However, the mechanisms underlying the anti-GBM properties of RA have not been fully elucidated. Epithelial to mesenchymal transition (EMT) and angiogenesis are important for the genesis and progression of GBM. These two crucial processes can be regulated by multiple molecular, including β-catenin, which has been demonstrated to act as a pro-tumorigenic molecular. In this study, we aimed to determine whether RA could suppress EMT and angiogenesis by inhibiting the action of β-catenin in GBM. We found that RA inhibited the proliferation, invasion and migratory properties of GBM cells. RA was also found to have downregulated the expressions of β-catenin and EMT-related biomarkers (N-cadherin, vimentin, and snail). In addition, the overexpression of β-catenin reversed the therapeutic effects of RA exerted on the EMT of GBM cells. RA restricted angiogenesis, as shown by the tube formation assay and CAM assay, while it downregulated VEGF levels in HUVECs. Moreover, massive β-catenin could reverse the suppression of angiogenesis induced by RA. Finally, we demonstrated that RA inhibited tumor growth and prolonged survival time *in* an intracranial U87 xenograft mouse model. Similar to the results *in vitro*, RA downregulated the expression of β-catenin, EMT makers and VEGF, and decreased vessel densit*y in vivo*. In summary, our results demonstrated that RA repressed GBM via downregulating β-catenin-mediated EMT and angiogenesis both *in vitro* and *in vivo*.

## Introduction

Due to high levels of morbidity and mortality, glioblastoma (GBM) has become the most common and malignant brain tumor which represents significantly high recurrence rate after optimal surgical resection and chemoradiotherapy treatment. Despite investigations made in developing more efficient methods of treatment, the prognosis of GBM patients remain poor due to its limitless proliferation, metastasis, radio-resistance and chemoresistance properties 1. Thus, new approaches for the treatment of GBM need to be urgently explored.

Increasing evidence has demonstrated that natural extracts obtained from traditional Chinese herbs display anti-cancer properties 2. Raddeanin A (RA), which is found in herbs of the *Ranunculaceae* family, is a pure active oleanane-type triterpenoid saponin, which has been reported to be able to suppress tumor growth in multiple cancer cell types, such as human gastric cancer, colorectal cancer and ovarian cancer, through the regulation of multiple signaling pathways 3-5. However, the effects and mechanism by which RA affects GBM have rarely been investigated.

Epithelial-mesenchymal transition (EMT), the process by which epithelial cells differentiate into mesenchymal cells, promotes GBM initiation and progression. During the EMT process, GBM cells acquire the characteristics of mesenchymal cells, which increases the motility of individual GBM cells and produces invasive behavior through the dissolution of epithelial junctions and the degradation of the extracellular matrix 6. Accumulating evidence has demonstrated that EMT plays a crucial role in the aggression of GBM 7. In addition, high expression levels of mesenchymal markers, such as N-cadherin, snail and vimentin, have been demonstrated in aggressive GBM and have been associated with the poor prognosis of GBM patients 8. Therefore, EMT inhibition is always an effective mode of treatment that can be used to decrease levels of progression and invasion of GBM. In previous studies, it was demonstrated that a variety of natural compounds could prevent cancers via anti-EMT 9. However, it has not been reported whether RA is capable of inhibiting EMT in GBM.

Angiogenesis is involved in tumor progression and invasion, and provides nutrients and blood for GBM metastasis and regeneration. Angiogenesis is communicated predominately through the expression of vascular endothelial growth factor (VEGF) to initiate tube structure formation in vascular endothelial cells, which leads to GBM neovascularization and malignancy 10, 11. Moreover, vascularization has been identified as a major characteristic of high-grade glioma, and has been found to be associated with GBM recurrence, which results in the generation of new blood vessels to create a hemopoietic microenvironment that leads to enhanced proliferation and distal glioma formation 12. Drugs that can suppress angiogenesis have potential to be used as methods of treatment for solid cancers 13.

β-catenin, a pivotal molecule of the Wnt/β-catenin signaling pathway, is highly expressed in a range of malignant tumors, including GBM. Additionally, β-catenin has been proved to function as a highly predictive biomarker of the poor survival of GBM patients 14, 15. Furthermore, β-catenin was found to exert various biological effects and regulated multiple biological phenomena, such as ontogenesis, apoptosis, and the cell cycle. It has also been reported that β-catenin is regarded as a pivotal hallmark of GBM and plays a vital role in EMT 16, 17. High level of β-catenin expression has been found to be involved in angiogenesis in GBM. Nuclear expression of β-catenin has been found to activate HIF-1α and cause an elevation in the expression of VEGF, which functions as an angiogenic factor in GBM vascularization 18, 19. Therefore, β-catenin is a potential target for GBM treatment. Recently, a study reported that RA could induce the apoptosis of human colorectal cancer cells by downregulating β-catenin expression 4. In our study, we aimed to explore the effects of RA on β-catenin expression in GBM.

The involvement of RA in GBM has not yet been fully elucidated. Therefore, our study pioneered investigations on the functional effects of RA on the decrease in EMT and angiogenesis and explored the role of β-catenin downregulation induced by RA in GBM. More importantly, using a mouse intracranial xenograft model we found that RA inhibited GBM growth through the suppression of invasiveness and angiogenesis *in vivo*. Therefore, this study not only identified RA as a potential agent but also laid the theoretical foundation for a novel approach for GBM treatment.

## Methods and Materials

### Chemicals, reagents, and antibodies

RA was obtained from Sigma-Aldrich (St. Louis, MO, USA). Antibodies used for western blotting analysis against β-catenin, N-cadherin, snail, vimentin, VEGF and GAPDH were purchased from Cell Signaling Technology (Beverly, MA), while VWF was purchased from Sigma-Aldrich (St. Louis, MO, USA).

### Cell lines and animals

The human GBM cell lines (T98G, LN299, U87, U251 and U87-luciferase) and primary human umbilical vascular endothelial cells (HUVECs) were obtained from the Chinese Academy of Medical Sciences (Beijing, China). The GBM cell lines were cultured in Dulbecco's modified Eagle medium supplemented with 10% FBS and 1% penicillin/streptomycin (Gibco, USA). The HUVECs were cultured in complete endothelial cell medium with 1% endothelial cell growth supplement (ECGS) and 10% fetal bovine serum (FBS) (Gibco, USA). All cell lines were cultured at 37°C in a humidified atmosphere containing 5% CO_2_. 6-week-old female BALB/c nude mice were obtained from Vital River Laboratories (Beijing, China) and kept in a controlled environment (23-25°C, 12 h light/dark cycles) approved by the Institutional Animal Care Committee of the Army General Hospital.

### Cell counting kit-8 (CCK-8) assays

The cells were seeded into 96-well plates at a density of 4× 10^3^ cells/well, followed by the addition of RA (0-800 nM) for 24 h. Then, CCK-8 solution was added into each well and incubated with the cells for 2 hours at 37°C, and absorbance was measured at 450 nm using a spectrophotometric plate reader.

### EdU assay

EdU (Guangzhou RiboBio) was added at 50 mmol/L and the cells were cultured for an additional 2 hours. After the removal of EdU containing medium, the cells were fixed with 4% paraformaldehyde at room temperature for 30 minutes, washed with glycine (2mg/mL) for 5 minutes in a shaker, treated with 0.2% Trion X-100 for 10 minutes, washed with PBS twice. Click reaction buffer was then added. After 10 to 30 minutes, the cells were washed with 0.5% Triton X-100 for 3 times, stained with DAPI for 10 minutes at room temperature, washed with 0.5% Triton X-100 for 5 times. Finally, immersed in 100 mL of PBS and examined under a fluorescence microscope.

### Cell migration and invasion assays

The cell migration and invasion assays were performed in 24-well Transwell Chambers (Corning, USA). The chambers were coated with (invasion) or without (migration) 100 µL of Matrigel (BD Biosciences, CA, USA). 2 × 10^4^ cells were placed in the upper chamber in 200µL serum-free media and the bottom chambers were filled with 600 μL of DMEM with 20% FBS. After 24 hours, the cells that had migrated into the lower chamber were stained with 0.1% crystal violet and five independent fields in each well were photographed.

### Capillary tube formation assays

The HUVECs were seeded into a 24-well plate precoated with Matrigel (BD Pharmingen, San Diego, CA), at a density of 1×10^4^ cells/well and were incubated with RA for the time durations indicated. The formation of capillary tubes in five low power fields were captured using a phase contrast microscope.

### Chorioallantoic Membrane (CAM) assays

The embryonic eggs were incubated in a humidified environment at 38 °C for 2 days and the shell membrane was stripped to obtain the chorioallantoic membrane, which had absorbed RA through the Whatman filter disk. Then, the hole was sealed, and the eggs were incubated for 48 h. The CAM was observed and the level of neovascularization was calculated under a microscope.

### Reverse transcription polymerase chain reaction (RT-PCR)

After the cells were treated with RA, total RNA was extracted using TRIzol reagent, according to the manufacturer's instructions (Sigma-Aldrich), and was subsequently used to reverse transcribe the RNA into cDNA, using the following primers: Human β-catenin: CACAAGCAGAGTGCTGAAGGTG (Forward), GATTCCTGAGAGTCCAAAGACAG (Reverse); Human GAPDH: TTGGTATCGTGGAAGGACTCA (Forward), TGTCATCATATTTGGCAGGTT (Reverse).

### Immunohistochemistry

The tumors were fixed using 4% paraformaldehyde and embedded in paraffin as they were collected. The tumors were deparaffinized and rehydration before being subjected to the immunohistochemical analysis. After treatment with 3% H2O2 for 10 min, the slides were blocked using 5% serum and incubated with primary antibodies overnight at 4°C and a secondary antibody labeled with HRP, then the slides were washed using PBS and were photographed and compared using Image Pro-Plus software.

### Transfection

For the overexpression of β-catenin, the cell lines were transfected with plasmids carrying β-catenin or flag only (GeneCopoeia, Maryland Rockville, USA) using a lipofectamine 3000 system, as instructed by the manufacturer.

### Western blotting

Cells lysates or xenograft glioblastoma tissue homogenates were lysed in 1× RIPA lysis buffer (CWBIO, China). All protein samples underwent separation by electrophoresis on 10% gradient gels in SDS-PAGE and blotted onto PVDF membranes by electroblotting in transfer buffer. Then, the membrane was put in 1× TBST containing 5% BSA for blocking for 1 hour at RT, then incubated overnight using primary antibodies suspended in 5% BSA (1×TBST) at 4 °C. After this, membranes were placed in incubation with peroxidase - conjugated secondary antibody and diluted in 1× TBST solution. The membrane was detected using ECL chemiluminescence method (Merck millipore, German ECL kit).

### RA treatment of the U87 xenograft mouse model

The animal protocols used were approved by the Institutional Animal Care Committee of the Army General Hospital. 6 week old female nude mice were injected intracranially with U87-luciferance cells (5×10^5^ cells/mouse) at a rate of 0.5 mL/min using a micro syringe pump (World Precision Instruments, Sarasota, FL, USA), which was attached to a Hamilton syringe with a 33-gauge needle (Hamilton, Reno, NV). The cells were injected into the mid-right striatum, using the following stereotaxic coordinates: 0.5 mm posterior, 2.8 mm ventral and 2.0 mm lateral of the bregma. 5 days after the cells were transplanted, tumor-bearing mice were divided into two groups (n = 5 each) and intraperitoneally injected with RA (100 mg/kg/day) or the vehicle. Tumor size and body weight were measured once every 10 days. At the end of these experiments, the mice were sacrificed, and the tumors were resected and homogenized to be used for western blotting analysis.

### Bioluminescence imaging

The mice were intraperitoneally injected with D-luciferin (Cambridge, MA) at a dose of 150 mg/kg of body weight and exposed to an IVIS Spectrum optical imaging system for 10 min to acquire bioluminescent images, which were analyzed to obtain signal intensities and were compared among the different groups using Living Image Software (Caliper Life Sciences).

### Statistical analysis

All data are presented as mean ± SD of experiments performed in triplicate. Differences between groups were examined using Student's t-test or ANOVA with LSD or Dunnett's T3 test performed using SPSS 20.0 software. The differences were considered significant if their p values were less than 0.05.

## Results

### RA inhibited the proliferation, migration, invasion abilities of glioblastoma cells

**Figure 1:**
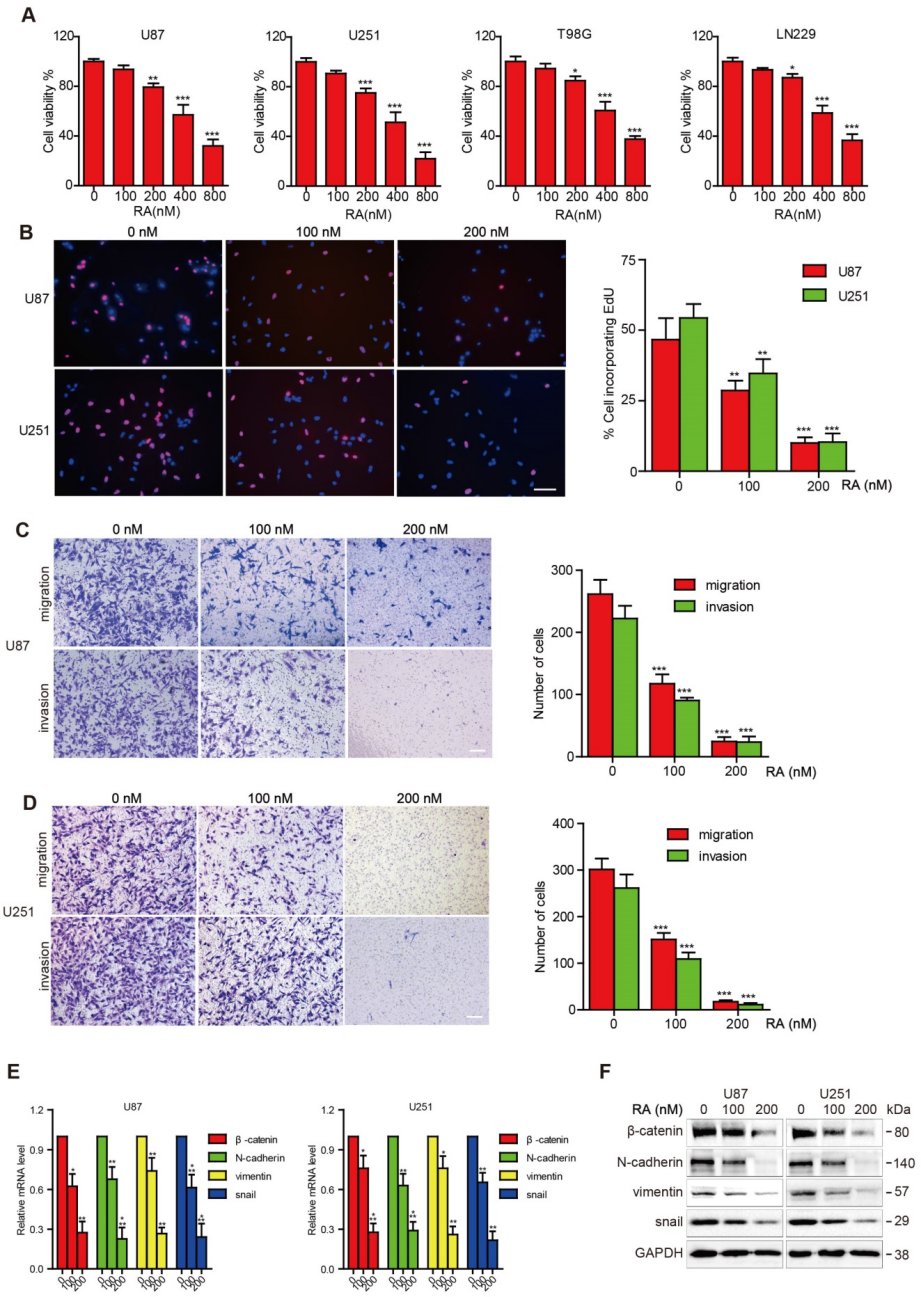
** RA suppressed tumor growth, migration, invasion and inhibited the expression of β-catenin and EMT-related proteins in GBM**. **(A)** The U87, U251, T98G and LN299 cell lines were treated with different concentrations of RA for 48 hours and then cell viability was determined using CCK-8 assay.** (B)** U87 and U251 cells were incubated for 48 hours with the indicated doses of RA and then EdU assay was performed. **(C, D)** U87 and U251 cells were treated with the indicated doses of RA for 24 hours, and then transwell assay was applied to determine the levels of migration and invasion. **(E)** qPCR and **(F)** western blotting analysis were performed on U87 and U251 cells treated with different doses of RA for 48 hours to analyze the mRNA and protein expression levels of β-catenin and EMT-induced proteins. n=3 or 4 and all tests were performed in triplicate. *, P < 0.05 compared with the control (0 μM).

First, we investigated whether RA could impose restrictions on GBM cell growth. RA inhibited the viability of the GBM cell lines (T98G, LN299, U87, U251) in a dose-dependent manner, as shown through the CCK-8 assays. The inhibition effect on T98G and LN299 cells caused by RA was weaker than that on U87 and U251 cells (Figure 1A). In addition, cell incorporation EdU assays confirmed the suppression effect of RA on the U87 and U251 cell lines (Figure 1B).

Next, we examined the effect of RA on migration and invasion. RA caused a significant decrease in the invasion and migration abilities of the cells (Figure 1C-D). These findings suggested that RA significantly decreased GBM cell proliferation, migration, and invasion abilities, which facilitate the aggressive behavior of GBM.

### RA inhibited the activity of β-catenin and EMT-related proteins in glioblastoma cells

In order to investigate the effect of RA on EMT transcription factors, we explored changes in β-catenin, N-cadherin, vimentin and Snail activity after treatment with RA. RA obviously abrogated the accumulation of β-catenin and EMT markers, N-cadherin, vimentin and Snail, both at transcription and translation levels (Figure 1E-F). These results revealed that β-catenin may participate in the disassembly of GBM MET through inhibition convergence of N-cadherin, vimentin, and snail activity caused by RA.

### Overexpression of β-catenin reversed the suppression of EMT in GBM cells caused by RA treatment

**Figure 2:**
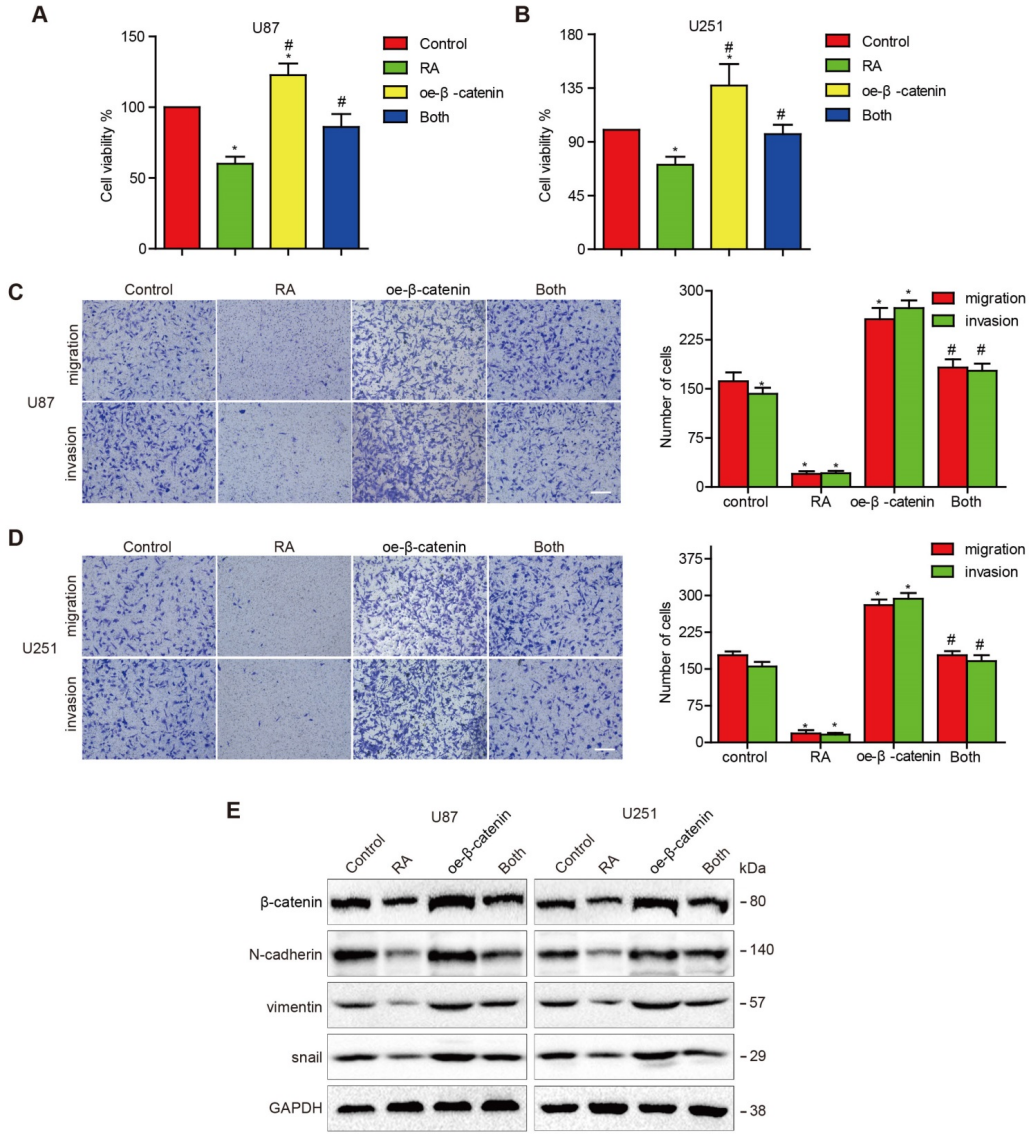
**Overexpression of β-catenin reversed the suppression of cell proliferation, migration, invasion and EMT induced by RA. (A, B)** U87 cells and U251 cells transfected with β-catenin or the vector plasmid for 18 hours were seeded into 96-well plates and incubated with 100 μM RA or the vehicle for 24 hours. CCK-8 assay was performed to determine the level of cell proliferation. (C, D) U87 cells and U251 cells were transfected with β-catenin or the vector plasmid and were seeded into a transwell chamber and examined using migration and invasion assays. (E) U87 cells and U251 cells transfected with β-catenin or the vector plasmid were then incubated with 100 μM RA for 48 hours. Western blotting analysis was performed to detect protein levels. *, P < 0.05 vs control; ^#^, P < 0.05 compared with either RA treatment or β-catenin transfection alone.

In order to further determine whether RA attenuated EMT in GBM cells via modulation of β-catenin activity, we treated U87 and U251 cells with transfected β-catenin plasmids to induce the overexpression of β-catenin. After the transfected cells were treated with RA, it was found that the overexpression of β-catenin abolished the inhibition effect on GBM cells induced by RA (Figure 2A-B) and similar outcomes were observed through the invasion and migration assays (Figure 2C-D). β-catenin overexpression continuously triggered the reduction of the effects induced by RA on N-cadherin, vimentin and Snail expressions (Figure 2E). Therefore, it was found that β-catenin plays a dominant role in the treatment effect induced by RA on GBM cells via suppression of proliferation and EMT.

### RA suppressed angiogenesis and downregulated VEGF

**Figure 3:**
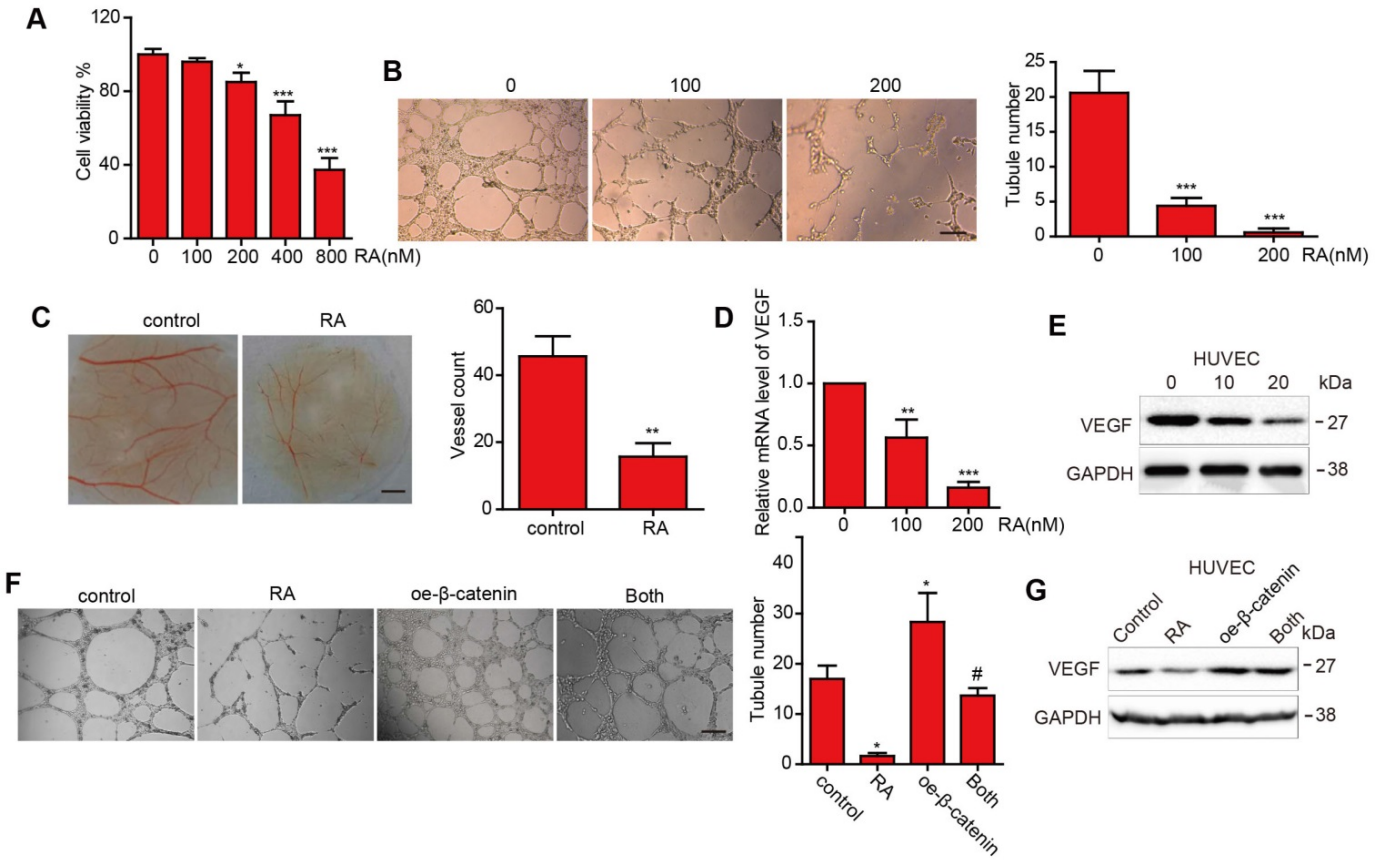
**The effect of RA on angiogenesis and VEGF expression in the HUVECs. (A-D)** HUVECs were treated with the indicated concentrations of RA. Then, (A) CCK-8 assay was used to determine cell proliferation. (B) Tube formation assay and CAM assay were applied to evaluate the angiogenic ability of the HUVECs. (D) Western blotting analysis was used to detect the protein level of VEGF. (E) Overexpression of β-catenin abolished the RA-induced anti-angiogenesis effect on the HUVECs. (F) Overexpression of β-catenin increased VEGF levels that had been decreased by RA treatment. *, P < 0.05 vs control; #, P < 0.05 compared with either RA treatment or β-catenin transfection alone.

First, we performed the CCK8 assay to estimate the proliferation inhibition effect in HUVECs (Figure 3A). Then we selected the low concentration (100nM and 200nM) to perform the next experiments to exclude the proliferation inhibition effect. To estimate the effect of RA on tubular formation, we used tube assay and found that RA significantly suppressed tubular formation in a concentration-dependent manner (Figure 3B). In addition, RA attenuated micro vessel in the CAM assay (Figure 3C). These results suggested that RA could inhibited angiogenesis *in vitro and vivo*. Next, we detected the VEGF protein level after treatment with the indicated concentration RA and found that RA downregulated VEGF in mRNA and protein levels in a dose-dependent manner (Figure 3D, E).

### Overexpression of β-catenin reversed the role of RA on angiogenesis and VEGF

To further investigate whether RA exerted anti-angiogenesis effect via β-catenin. We overexpressed β-catenin plasmid or its vector plasmid followed by with or without RA treatment. And we found that overexpression of β-catenin increased angiogenesis and VEGF protein expression, and overexpression of β-catenin abolished the effects of RA on angiogenesis and VEGF upregulation in HUVECs (Figure 3F, G).

### RA decreased glioma growth in an orthotopic xenograft mouse model

**Figure 4:**
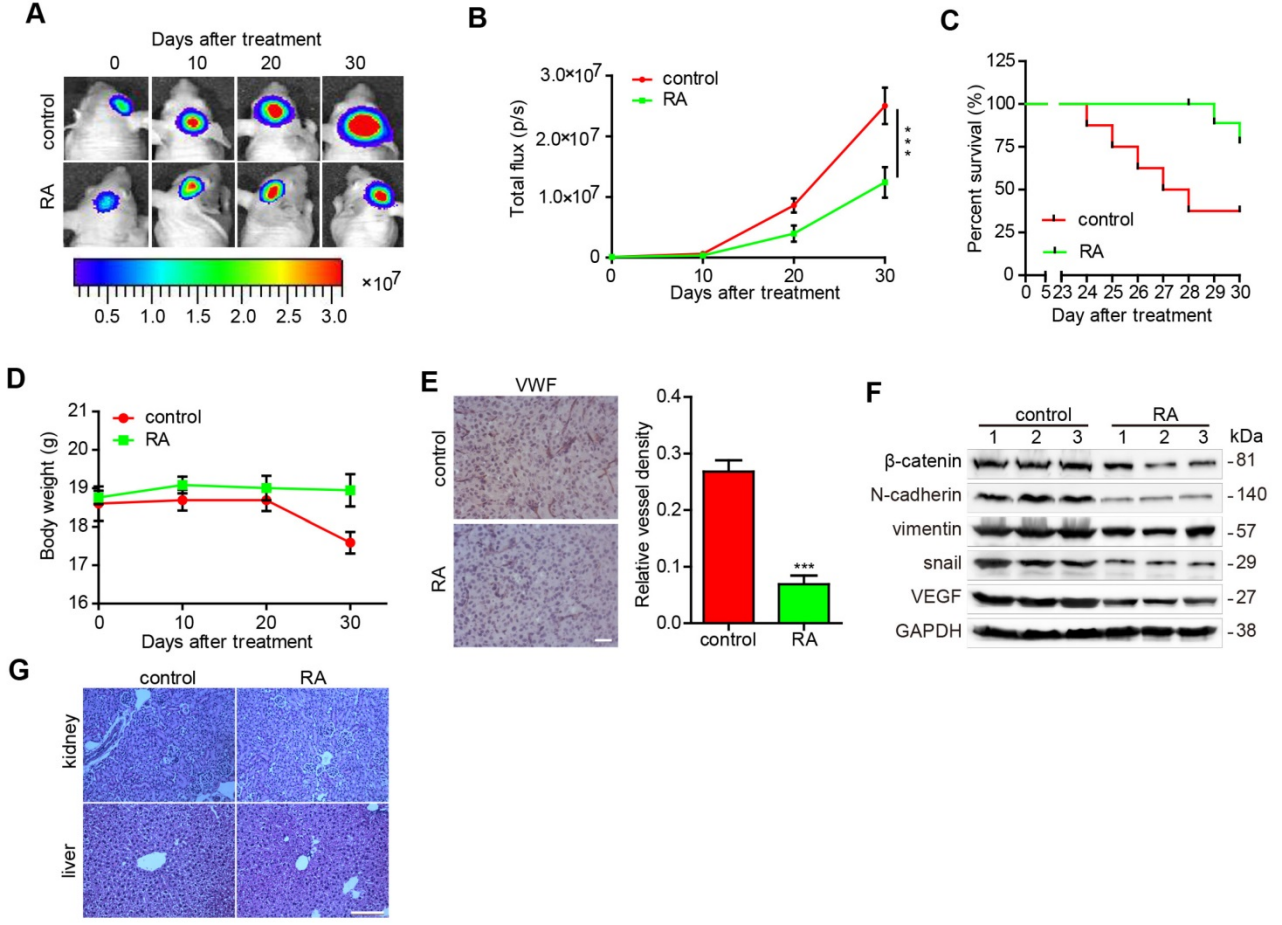
** RA decreased glioma growth in an orthotopic xenograft mouse model** U87-luciferace cells (5×10^5^) were intracranially injected into the mid-right striatum of 6-weeks-old male BALB/c nude mice. 5 days after injection, tumor formation was examined using bioluminescence imaging and the mice were divided into two groups: mice that were intraperitoneally injected with the vehicle (control) and mice that were intraperitoneally injected with RA (100 mg/kg/day). Tumor sizes were measured once every 10 days. Bioluminescence imaging was used to measure tumor volume. **(A)** The tumor volume of mice in each group determined at each time point **(B)** Tumor volume of mice in each group determined at each time point **(C)** The survival rate of mice in each group **(D)** The body weight of the mice was measured at the time points indicated. At the end of the experiment or after the mice had died, their brains were collected. **(E)** VWF (Bar, 50 μm) were used to stain the mice brains. Images of the brain were captured and analyzed using Image Pro-Plus software. **(F)** At the end of the experiment or after the mice had died, tumor tissues were collected from the mice and the protein lysates obtained were examined to determine levels of protein expression. **(G)** the kidney and liver tissues were obtained and HE-stained (Bar, 500μm). ***, P < 0.001, compared with the control.

Finally, we explored the function of RA using a GBM orthotopic xenograft mouse model established using intracranial U87-luciferase labeled cells. As illustrated in figure 4A and 4B, tumor volumes were found to have decreased after RA treatment in a time-dependent manner, as shown by the intensity of luciferase activity, compared with that of the blank groups (Figure 4A-B). In addition, mice with RA achieved a higher survival rate and inconspicuous change in weight (Figure 4C-D). Furthermore, we also observed that the density of vessels in the RA groups were evidently lower than that of the blank groups (Figure 4E). Moreover, EMT and angiogenesis factors in the U87 xenograft mouse brains were detected and RA treatment was found to have diminished the expression of EMT (β-catenin, N-cadherin, vimentin and Snail) and vascular (VEGF) markers (Figure 4F). Our results suggested that RA also suppressed tumor growth and inhibited EMT and angiogenesis *in vivo*. To evaluate the safety of RA *in vivo*, we then obtained kidney and liver tissues from the above groups and conducted HE staining. And we noted no clear pathological changes in the kidney and liver tissues of the two groups (Figure 4G). These findings suggest that RA is safe when administered *in vivo*.

## Discussion

Despite the availability of treatment methods that combine surgical resection with chemoradiotherapy, it is still not possible to completely cure GBM or remove tumors in order to prolong patient survival due to the unlimited growth and invasiveness resulting from EMT and angiogenesis. Hence, we have struggled to identify natural products used in traditional Chinese medicine that may help achieve enhanced levels of efficacy. In this study, we confirmed that RA, an active triterpenoid saponin, inhibited EMT and angiogenesis through the downregulation of β-catenin expression.

RA is a natural compound extracted from *Anemone raddeana* Regel that has been shown to exert anti-cancer activity in several types of tumors. It has been reported that RA inhibited the progression of human colorectal, osteosarcoma, prostate and breast cancer 3-5, 20. However, little research has focused on the capacity of RA to be used for the treatment of GBM. In our study, as shown in figure 1A and 1B, RA was found to significantly decrease the growth of four GBM cell lines in a concentration-dependent manner. This result is consistent with that of a previous study by Peng et al., which showed that RA inhibited GBM U87 and U251 cell growth via inducing apoptosis 21. Our study confirmed that RA exerts an inhibitory effect on GBM.

Among them, EMT and angiogenesis are the two most important processes in the initiation and progression. VEGF is a direct inducer of revascularization and it has been reported that VEGF collaborates with Snail to form a positive feedback loop that enhances cancer progression and invasion 22, 23. In previous studies, RA has been demonstrated to induce apoptosis, inhibit invasion, promote autophagy and suppress proliferation 3-5, 20. And Xue et al. reported that RA upregulated the epithelial marker, E-cadherin, in human gastric cancer cells and suppressed invasion *in vitro* 24. Moreover, Guan et al. reported that RA decreased the tube formation in HUVECs 25. In the current study, we demonstrated that RA downregulated mesenchymal markers (N-cadherin, snail and vimentin) in GBM. In addition, Guan et al reported that RA has been shown to inhibit the angiogenesis in human colorectal cancer cells 25. In our study, we verified that RA not only decreased the tubulation ability and VEGF expression of HUVECs but also that it inhibited angiogenesis, as shown through the CAM assay. We also confirmed that RA suppressed angiogenesis in an orthotopic murine glioma model. Our research further confirmed the inhibition ability of RA on angiogenesis in GBM *in vitro* and *in vivo*, which provided new insights into the potential of RA for GBM treatment.

β-catenin is a crucial regulator that is strongly associated with EMT-induced deadhesion and movement. Nuclear localization of β-catenin results in binding with the promoter that encodes for EMT genes and promotes the transcription and translation of N-cadherin, vimentin and Snail 17, 19. Moreover, β-catenin is required for angiogenesis 26. Additionally, Wang et al. have reported that RA inhibited growth and promoted apoptosis in human colorectal cancer by suppressing β-catenin expression 27. In this study, we reported that RA suppressed EMT and angiogenesis by downregulating β-catenin expression in GBM.

Most agents that exert an anti-GBM effect *in vitro* remain ineffective and even if a drug can inhibit tumor growth in a subcutaneous GBM model, it may fail to suppress GBM in CNS due to the existence of the blood-brain barrier (BBB). In previous investigations, RA was always applied *in vitro* or away from the CNS. However, in our study, we verified the anti-EMT and antiangiogenic properties of RA in a GBM orthotopic xenograft mouse model. RA commendably decreased the tumor volume and facilitated cell death without an obvious change in the weight of the mice. Additionally, RA negatively-regulated the expression of β-catenin, EMT-induced proteins (N-cadherin, vimentin, and Snail) and VEGF protein in the orthotopic xenograft mouse model.

Overall, we found that RA was able to inhibit angiogenesis and EMT in GBM by downregulating β-catenin expression *in vitro*. We also demonstrated, for the first time, that RA exerted anti-GBM properties *in* an intracranial glioma model, which was consistent with its mechanism of action *in vitro*. Therefore, RA may be a high potential therapeutic drug for GBM treatment in clinical.
